# Delphi consensus on the diagnosis and treatment of patients with short stature in Spain: GROW-SENS study

**DOI:** 10.1007/s40618-021-01696-0

**Published:** 2021-11-17

**Authors:** R. Corripio-Collado, C. Fernández-Ramos, I. González-Casado, F. Moreno-Macián, J.-P. López-Siguero, J.-I. Labarta-Aizpún

**Affiliations:** 1 Paediatric Endocrinology Unit, Hospital Universitario Parc Taulí, Sabadell, Barcelona Spain; 2 Paediatric Endocrinology Unit, Hospital Universitario Basurto, Bilbao, Spain; 3 Pediatric Endocrinology Deparment, Hospital Universitario La Paz, Madrid, Spain; 4 Paediatric Endocrinology Unit, Hospital Universitario y Politécnico La Fe, Valencia, Spain; 5 Paediatric Endocrinology Unit, Hospital Regional Universitario de Málaga, Instituto de Investigación de Málaga (IBIMA), Malaga, Spain; 6 Paediatric Endocrinology Unit, Paediatric Department., Hospital Universitario Miguel Servet, Instituto de Investigación Sanitaria de Aragón, Zaragoza, Spain; 7 Department of Microbiology, Pediatrics, Radiology, and Public Health. School of Medicine, Zaragoza University, Avenida Isabel la Catolica 1-3, 50009 Zaragoza, Spain

**Keywords:** Adherence, Delphi, Diagnosis, Growth hormone, Monitoring, Short stature

## Abstract

**Purpose:**

To identify consensus aspects related to the diagnosis, monitoring, and treatment of short stature in children to promote excellence in clinical practice.

**Methods:**

Delphi consensus organised in three rounds completed by 36 paediatric endocrinologists. The questionnaire consisted of 26 topics grouped into: (1) diagnosis; (2) monitoring of the small-for-gestational-age (SGA) patient; (3) growth hormone treatment; and (4) treatment adherence. For each topic, different questions or statements were proposed.

**Results:**

After three rounds, consensus was reached on 16 of the 26 topics. The main agreements were: (1) diagnosis tests considered as a priority in Primary Care were complete blood count, biochemistry, thyroid profile, and coeliac disease screening. The genetic test with the greatest diagnostic value was karyotyping. The main criterion for initiating a diagnostic study was prediction of adult stature 2 standard deviations below the target height; (2) the main criterion for initiating treatment in SGA patients was the previous growth pattern and mean parental stature; (3) the main criterion for response to treatment was a significant increase in growth velocity and the most important parameter to monitor adverse events was carbohydrate metabolism; (4) the main attitude towards non-responding patients is to check their treatment adherence with recording devices. The most important criterion for choosing the delivery device was its technical characteristics.

**Conclusions:**

This study shows the different degrees of consensus among paediatric endocrinologists in Spain concerning the diagnosis and treatment of short stature, which enables the identification of research areas to optimise the management of such patients.

**Supplementary Information:**

The online version contains supplementary material available at 10.1007/s40618-021-01696-0.

## Introduction

Short stature is one of the main reasons for paediatric consultation [1]. Hence, the importance of making a correct assessment allows the diagnostic process to be properly directed. Although there is no international consensus defining normal versus pathological growth, the most frequently used criteria for short stature or abnormal growth are as follows: (a) height below − 2 standard deviations (SD) for age and sex on growth curves corresponding to the population studied; (b) normal height (between ± 2 SD), but 2 SD below target height; (c) estimated adult stature below − 2 SD of target height; and (d) reduced growth velocity, below − 1 SD (25th percentile) for age and sex, maintained over a period of 1 year or, in the absence of short stature at a growth velocity less than − 2 SD [1–5]. The choice of appropriate growth curves is very important in growth assessment and the use of updated national curves is recommended [6–8].

There is different terminology used to classify short stature, but, in general, all of it can be divided into pathological or known-cause short stature and idiopathic or unknown-cause short stature [1, 5]. Idiopathic short stature has been extensively reviewed, and its concept has been debated and discussed in recent years. Differentiation between idiopathic short stature and idiopathic growth hormone (GH) deficiency is often difficult and reflects the poor discriminating power of somatotropic axis tests, since many patients diagnosed with short stature are variants of normal growth [1, 5].

Since there is little uniformity in the criterion for management of short stature, this study has been proposed to evaluate clinical practice in the management of short stature in Spain and show the degree of consensus in the management of this pathology.

## Methods

### Study design

The GROW-SENS study was conducted using a modified Delphi method of quantitative, semi-quantitative, and qualitative analyses. This is a structured, group communication technique whereby written online surveys can be conducted to collect and unify the opinions of a group of experts about a complex or controversial topic with insufficient scientific evidence. This technique avoids the difficulties and drawbacks inherent to consensus methods based on face-to-face discussion, such as travel, influence biases, and non-confidential interaction [9, 10].

The process was carried out in four phases: (1) creation of a scientific committee in charge of the project; (2) kick-off meeting to propose the main topics through a review of the latest literature evidence; (3) three successive rounds of online surveys to gather the opinions of a panel of experts; and (4) analysis of the results and discussion of the conclusions by the scientific committee.

### Participants

The scientific committee coordinated the entire consensus and was made up of six national experts in paediatric endocrinology. The panel of participating experts was selected by the scientific committee and included 56 paediatric endocrinologists from different Spanish provinces. The set of panellists had a minimum of 10 year experience as paediatricians mainly specialising in paediatric endocrinology and they were all members of the Spanish Paediatric Endocrinology Society (SEEP). The panellists were invited to participate in the study by e-mail, informing them of the nature of the study through a presentation letter.

### Questionnaire

At the kick-off meeting, the scientific committee discussed the main strategies for the diagnosis and treatment of short stature. As a result of this meeting, 26 topics were proposed, grouped into four blocks: (1) diagnosis; (2) monitoring of the small-for-gestational-age (SGA) patient with short stature; (3) GH treatment; and (4) treatment adherence. For each block, several questions or statements were proposed which, depending on the type of response, could be dichotomous, multi-response, qualitative, quantitative, and, often, Likert scale.

The Likert scale used consisted of 11 items grouped as follows: 0–2, interpreted as not at all important or strongly disagree; 3–4, interpreted as slightly important or disagree; 5–6, interpreted as important or neither agree nor disagree; 7–8, interpreted as very important or agree; and 9–10, interpreted as extremely important or strongly agree.

### Delphi consensus

Consensus was reached in three rounds of consultations held between March 2019 and March 2020. In the first round, the participants answered the online questionnaire with the possibility of adding their opinion with open text. Following analysis of the answers provided, the questions were rephrased in the second round and then again in the third round. The results of the three rounds were tabulated and presented in a descriptive form to be analysed, interpreted, and discussed jointly by the scientific committee to find common ground and provide useful conclusions and recommendations for the clinical practice of paediatric endocrinologists.

### Analysis and interpretation of the results

The data analysed provided semi-quantitative information (scores of the responses collected through Likert scales) and qualitative information (analysis of the experts' discourse in open-ended questions). A minimum of 70% of homogeneous responses was established to consider that there was consensus among the panel of experts on the answer to this question.

### Ethical considerations

All the recommendations set out in the Helsinki declaration were used. All participants gave informed consent and no personal information was recorded at any time. To preserve the confidentiality of the opinions, all information was coded.

## Results

Out of the 56 endocrinologists recruited, 43 completed the first round, 37 completed the second round, and 36 completed all three rounds of the study. Out of the 26 items proposed, consensus was reached on 16 of them (61.5%). Tables 1, 2, 3, and 4 show the topics dealt within each block and the degree of consensus reached on those that exceeded 70%.

**Table 1:** Diagnosis of short stature

Blocks and topics	Statements	Consensus*	
		Yes (> 70%) (%)	No (< 70%) (%)
1. Diagnostic criteria for short stature	If a growth rate ≤ − 1 SD is identified for more than 1 year, that is enough time to initiate a growth stunting study	76	
	Prediction of adult height 2 SD below the target stature	94	
2. Growth curves for diagnosis of short stature	Most suitable growth curve: Spanish Cross-sectional Study 2010	72	
3. Need for a national study to assess the current reference height of the population in Spain	In favour		56
	Not necessary		44
4. Calculation of the adult height prognosis established from bone age	Bone age is a useful criterion to calculate the adult stature prognosis	89	
5. Tests considered to be of priority use in Primary Care	Complete blood count Biochemistry: blood sugar level, renal function, hepatic tests, venous blood gas analysis (< 3 years old), Ca and P, Na and K Thyroid function Coeliac disease screening	100	
6. Diagnostic value of karyotyping and other genetic studies in children with short stature	Karyotyping provides significant value for diagnosis	98	
	The *SHOX* gene study is useful when the following criteria are present: Familial short stature with autosomal dominant inheritance pattern Abnormality of body proportions Suggestive radiological findings	80	
	Genetic study of bone dysplasias is rarely or never carried out	76	
	Exome sequencing study is rarely or never carried out	89	
7. GH stimulation tests considered key to determining the diagnosis of such a hormone deficiency	GH stimulation is not a decisive test for the diagnosis of GH deficiency	86	
8. Tests necessary to demonstrate an isolated GH deficiency and those used in clinical practice	Two tests are not considered necessary to diagnose isolated GH deficiency	72	
9. Appropriate cut-off point for establishing a diagnosis of GH deficiency	< 10 ng/m		14
	< 7 ng/ml		56
	< 5 ng/ml		2
	Other		28
10. Gonadal steroid priming at peripubertal ages to establish a diagnosis of GH deficiency	Not useful or necessary		56
	Necessary		44
11. Other conditions that may cause short stature in which it is considered that GH deficiency testing should be performed	Chronic inflammatory diseases		47
	Corticodependence		30
	Cystic fibrosis		21
	Attention deficit hyperactivity disorder		19
	Others		40
12. Need for reassessment of GH deficit at adult height	Re-evaluation of GH deficiency at adult height is not considered necessary if IGF-I levels are measured in isolated idiopathic GH deficiency	74	
13. Attitude towards a 13-year-old male with 3 ml testicular volume and decreased growth velocity (less than − 1 SD)	Conduct tests without priming		38
	Wait for 8 ml of testicular volume		35
	Perform functional tests with priming		27

*SD* standard deviations, *GH* growth hormone

*There is considered to be consensus when more than 70% homogeneous responses have been recorded

**Table 2:** Small-for-gestational-age patient

Blocks and topics	Statements	Consensus*	
		Yes (> 70%) (%)	No (< 70%) (%)
1. Information that should be essential for initiating GH treatment in SGA patients	Length and weight at birth	95	
	Height at 4 years	94	
	Previous growth pattern	100	
	Average parenteral size	97	
2. Silver–Russell syndrome as an exclusion criterion for initiating GH treatment in SGA patients	It is not considered an exclusion criterion	88	
3. Appropriate age for initiation of GH treatment in the non-recovering SGA patient	From the age of 2 years, if the GH indication is authorised at that age		54
	From the age of 4 years, even if there is an indication from the age of 2 years		46

*SD* standard deviations, *GH* growth hormone

^*^There is considered to be consensus when more than 70% homogeneous responses have been recorded

**Table 3:** Growth hormone treatment

Blocks and topics	Statements	Consensus*	
		No (< 70%) (%)	No (< 70%) (%)
1. Most appropriate age for commencement of GH treatment in a child with Prader–Willi syndrome	Before 2 years old		67
	Between 1 and 2 years		42
	From 2 years old		33
2. Attitude towards a patient who does not respond to treatment	The sequence that reflects a better attitude on the part of the specialists is: 1. Check treatment adherence 2. Increase the dose 3. Evaluate the diagnosis 4. Assess comorbidities 5. Discontinue treatment	76	
3. Attitude towards the approach to a child with GH deficiency in the context of an oncological history: time considered appropriate to wait to start GH administration from the onset of remission, assuming that there are no contraindications and that the oncologist and parents agree	Preferably before 2 years old		34
	Preferably after 2 years old		66
4. Recommended tests, in addition to thyroid function, bone age and IGF-I, to monitor for adverse events that may arise from GH treatment	Carbohydrate metabolism test	100	
	Lipid metabolism test	78	
5. Most important parameters in the assessment of response to GH treatment according to indications for treatment of short stature	Significant increase in growth velocity	73	
6. Freedom of GH prescription	I can choose between two or three options		50
	It is up to the administration to decide		28
	I have freedom of prescription		22

*SD* standard deviations, *GH* growth hormone

*There is considered to be consensus when more than 70% homogeneous responses have been recorded

**Table 4:** Treatment adherence

Blocks and topics	Statements	Consensus*	
		Yes (< 70%) (%)	No (< 70%) (%)
1. Criteria for personalised choice of a GH delivery device	Technical characteristics of the device	97	
	Drug data sheet	73	
	Hospital criteria	70	
	User preferences	70	
2. Resources for monitoring GH treatment adherence	Use of recording devices	97	
	Health education for children and parents	95	
	Nursing support	86	
	New technologies: SMS, Apps, e-Health	84	
3. Priority resources for improving GH treatment adherence	1st sequence: health education; nursing support; use of recording devices; new technologies		50
	2nd sequence: health education; use of recording devices; nursing support; new technologies		50
4. Influence of improved child self-esteem as a factor that favours GH treatment adherence	The child's self-esteem is a factor that favours GH treatment adherence	94	

*SD* standard deviations, *GH* growth hormone

*There is considered to be consensus when more than 70% homogeneous responses have been recorded

### Block 1: diagnosis of short stature

Out of the 13 proposed topics on the diagnosis of short stature, consensus was reached on 8 of them (61.5%). The topic with the highest degree of consensus (100%) was tests considered to be of priority use in Primary Care. Participants agreed that the tests should be complete blood count, biochemical analyses, thyroid function, and screening for coeliac disease.

Another topic with a high degree of consensus was the diagnostic value of genetic testing. 98% of the specialists considered karyotyping to be of important diagnostic value. The *SHOX* gene sequencing study was considered an additional test by 80% of panellists. Exome sequencing and study of bone dysplasias are never performed or are infrequent according to 89% and 76% of respondents, respectively. 65% agreed that performing genetic testing for Noonan syndrome is very rare or never performed.

Among the diagnostic criteria for study of short stature with the highest degree of consensus are the prediction of adult stature 2 SD below the target height (94%) and a growth velocity ≤ − 1 SD for more than 1 year (76%). There was consensus on the method of how the calculation of a patient's adult stature prognosis is performed, with the majority of specialists (89%) mentioning that they use bone age to establish it. 72% of the experts use the Spanish Cross-sectional Growth Study 2010 as a reference standard.

A high percentage of the specialists (86%) did not agree that GH stimulation tests are a decisive test in the diagnosis of GH deficit and 72% consider that it is not necessary to perform two tests to diagnose an isolated GH deficit.

No consensus was reached concerning the following topics: most appropriate cut-off point to establish a diagnosis of GH deficit; the need to perform, at this time, a national growth study to establish the reference standards for the Spanish population; use of gonadal steroid priming testing at peripubertal ages; the approach that should be taken in a 13-year-old male patient with a testicular volume of 3 ml and decreased growth velocity; and the clinical conditions that may cause short stature under which a GH deficiency study should be performed.

The results obtained from the scores the panellists gave to the statements proposed in block 1 are presented in Table 1.

### Block 2: small-for-gestational-age patient

Regarding the block on monitoring the SGA patient, consensus was reached on two of the three topics proposed (66.7%). The main criteria considered indispensable for starting GH treatment in these patients were: length and weight at birth (95%), stature at 4 years (94%), previous growth pattern (100%), and mean parental stature (97%).

In SGA patients, most specialists (88%) did not consider Silver–Russell syndrome to be an exclusion criterion for initiating GH treatment. There was no consensus concerning the appropriate age for the initiation of GH treatment in the non-recovering SGA patient.

The results obtained from the scores the panellists gave to the statements in block 2 are presented in Table 2.

### Block 3: GH treatment

Of the 6 proposed topics on GH treatment, consensus was reached on 3 of them (50%). For monitoring of adverse events resulting from GH treatment, in addition to thyroid function tests, bone age, and IGF-I levels, the participants stated that the most important tests are the assessment of carbohydrate metabolism (100%) and lipid metabolism (78%).

Regarding the attitude towards a non-responding patient, the most recommended sequence of action (76%) was: (1) check treatment adherence; (2) increase the dose; (3) reconsider the diagnosis; (4) assess comorbidities; and (5) discontinue treatment.

The parameter with the highest consensus for assessing response to GH treatment in patients with short stature was a significant increase in growth velocity (73%).

Only 22% of participants are free to prescribe any GH, 50% can only choose between two or three options, and for 28%, the administration chooses the treatment.

There was no consensus concerning either the age for the initiation of GH treatment in children with Prader–Willi syndrome or the waiting time for an oncological patient to start GH treatment after the start of remission.

The results obtained from the scores the panellists gave to the statements in block 3 are presented in Table 3.

### Block 4: treatment adherence

In the block on treatment adherence, out of the four topics proposed, consensus was reached on three. Regarding the criteria for a personalised choice of GH delivery device, the specialists indicated the following as the most important: technical characteristics of the device (97%), drug data sheet (73%), hospital criteria (70%), and user preferences (70%).

The resources that specialists considered most important for monitoring adherence to GH treatment were: use of electronic recording devices (97%), health education (95%), nursing support (86%), and new e-health technologies (84%).

94% considered the child's self-esteem to be an element favouring adherence.

There was no consensus concerning the prioritisation of resources to monitor treatment adherence.

The results obtained from the scores the panellists gave to the statements in block 4 are presented in Table 4.

## Discussion

This is the first study in Europe with the Delphi method to assess the diagnosis and management of patients with short stature during childhood. The degree of consensus reached in this study was high: for 16 of the 26 topics proposed, it was higher than 70%. Despite the existence of some expert consensus [1, 5], many of the topics related to the diagnosis and treatment of short stature are still a matter of debate. The GROW-SENS study was designed with the aim of finding out the opinion of paediatric endocrinologists on these aspects. The Delphi method used in this paper in relation to short stature and GH is novel in this field.

### Block 1: diagnosis of short stature

The prevalence of pathological short stature in children referred for assessment is around 5% and varies between 1.3% and 19.8%. There is significant variability in establishing when to initiate a short stature study [11–13]. There is no evidence-based consensus that determines the tests that must be performed in a study of a child with short stature [5] and the diagnostic yield of the tests used in children with short stature is very low [14].

Different authors propose conducting a karyotype in girls with short stature with an unexplained cause, shorter than their genetic stature or when they present two or more dysmorphic characteristics [15–18]. Within the scope of Primary Care, it is more common to request a complete blood count, general biochemical parameters, thyroid function, and screening for coeliac disease. It is estimated that 2–8% of children with non-familial short stature without gastrointestinal symptoms have coeliac disease [19–21], and this is in agreement with the findings of the study performed.

There was controversy as to whether it is advisable to request IGF-I serum levels in Primary Care, due to the difficulties of interpretation and the methodological variability involved. Determination of IGF-I should be the first test to be performed in Specialised Care to study the somatotropic axis, since an IGF-I higher than the 50th percentile associated with a growth velocity higher than the 25th percentile makes a GH deficit highly unlikely [19, 22, 23].

Regarding the request for genetic tests other than karyotyping, study of the *SHOX* gene was the most requested by the participants, especially in the case of familial short stature with autosomal dominant inheritance pattern, abnormalities in body proportions, and/or suggestive radiological findings.

It should be noted that assessments expressed by participants concerning each genetic test reflect the frequency with which those tests are requested, which may be influenced not only by clinical judgement, but also by availability. The benefit of genetic studies to diagnose short stature increases when they are associated with systematic phenotyping of patients [24].

Regarding the criteria for diagnosing short stature, participants clarified that an isolated height measurement is not sufficient and needs to be verified by successive calculations. It was also clarified that although an extreme growth velocity and short stature (below − 2.5 SD) with poor prognosis for adult stature are two important criteria for the diagnosis of pathological short stature, if the parents' values are very different, this indicator loses sensitivity.

Most of the specialists interviewed considered that the most appropriate growth curve is that in the Spanish Cross-sectional Study 2010 [25, 26]. In spite of this, some of the panellists said that other Spanish curves are also occasionally used [6, 27].

Although it was not considered a priority to carry out a national study to assess the reference stature of the Spanish population, the general recommendation is to do so every 10–15 years, especially due to the increase in the different ethnic groups that live in Spain [28]. In fact, there are few studies on the immigrant population in Spain.

Many of the participants did not agree that GH stimulation was decisive for their diagnosis. However, they considered this test to be of greater value in ruling out a deficit than in confirming it, i.e., it has greater specificity than sensitivity. There are studies in which a level below 3 ng/ml may correlate with severe deficits [1]. However, despite new laboratory techniques, no exact cut-off point has been established. Lack of uniformity in the criteria to establish the usefulness and indication of stimulation tests, as well as the cut-off level necessary to diagnose a GH deficit, has been pointed out by various authors internationally [29–31]. Stimulation tests remain valid for diagnosis of GH deficit, but one must consider that the measured GH concentration varies depending on the type of test used and the immunoassay employed, so they must be interpreted together with the patient's auxology.

In any case, in general, the study of GH deficiency should be considered in all pathologies with an alteration in growth velocity that cannot be explained for any other reason.

Gonadal steroid priming to establish the diagnosis in prepubertal ages remains a controversial topic. There is little evidence in studies that support its use, since the group of patients analysed is small and the patients included have different characteristics in terms of chronological age, auxology, and pubertal development. The absence of large, homogenous series in terms of the patients' characteristics would explain why just 30–40% of paediatric endocrinologists use it before studying GH secretion. In any case, it must be performed and interpreted on an individual basis [1, 32, 33].

With regard to reassessment of GH deficit in adolescence when adult stature is reached, 74% of the experts considered that reassessment is not necessary in cases of isolated GH deficit with levels of IGF-I within normality. This finding contrasts with a Delphi study performed by the Italian Endocrinology Society in which there is consensus regarding the need for reassessment of these patients [34].

In summary, diagnosis of a GH deficit in childhood and adolescence should be based on a combination of factors that include auxology, and radiological and laboratory evaluation, together with clinical experience [35].

### Block 2: small-for-gestational-age patient

Birth length or birth weight was considered to be the most indispensable criteria for initiating GH treatment in SGA children. However, it was clarified that cases in which this is not known need to be individualised. Furthermore, although it was agreed that parental mean stature could be a criterion for initiating treatment, it was argued that it should not be a limiting factor for initiating treatment in SGA children who meet all other inclusion criteria and Silver–Russell syndrome should not be an exclusion criterion for initiating GH treatment [36, 37]. The lack of consensus on the appropriate age to initiate GH treatment may be due to the lack of evidence of superiority of response for early initiation of treatment. Even so, it has been reported that 90% of SGA children can experience a catch-up growth spurt that mostly takes place in the first 12 months of life and is completed by 2 years of age, reaching a stature greater than − 2 SD [38, 39].

### Block 3: GH treatment

To monitor adverse events arising from GH treatment, in addition to thyroid function tests, bone age, and IGF-I levels, the need to check carbohydrate metabolism was identified, with lipid metabolism being less important [33]. However, other tests to be performed on an individual basis were also mentioned, such as orthopaedic examinations and fundus evaluation due to clinically suggested suspicion of endocranial hypertension, etc.

The response to GH treatment can be assessed with the increase in stature and growth velocity, expressed in SD or cm/years [40]. The response in the first year of treatment is considered very important due to its high predictive value for gain in adult stature [41]. It was proposed to assess patients' quality of life as an important response parameter, although its quantification may be heterogeneous due to the fact that standardised quality-of-life questionnaires for children with short stature are rarely used in clinical practice.

67% of respondents would initiate GH treatment in children with Prader–Willi syndrome before the age of 2 years, since it has been shown that GH is safe at that age [42]. It is essential to have contact with expert centres with greater experience that can assist in the use of GH in these patients.

There is lack of consensus on the waiting time before administering GH in a child with a deficit in oncological remission.

### Block 4: treatment adherence

Treatment adherence conditions the response and efficacy of the treatment and is a common problem in all chronic diseases that require maintained treatment [43]. Poor adherence implies a loss of stature, but there are very few studies that have quantified this and it continues to be discussed whether the degree of adherence differs according to the indication [44]. In the case of a non-responder patient, the attitude chosen first is to check treatment adherence. To monitor adherence, the most valued resource was the electronic record, although it was made clear that this should not replace health education, but it is evident that the same degree of objectivity cannot be achieved through any other method. However, the indications for use in the drug's technical data sheet should be taken into account.

Given the importance of the child's self-esteem for good treatment adherence, this should be measured and recorded as a parameter through scales validated for this purpose, although they are rare in clinical practice.

### Limitations of the study

Meetings of experts are, per se, a limitation in terms of the level of evidence. However, out of the more than 200 members of the SEEP, the members with more than 10 year experience in using GH were selected.

The participation of members from a country with a predominantly publicly financed health system is another of the study's limitations.

## Conclusions and future prospects

With regard to the implications for everyday clinical practice, and in spite of the heterogeneity of some topics, this study will provide novice paediatric endocrinologists with a guide for critical decisions on the use of GH in childhood.

In terms of future research, it is necessary to verify our results with a larger sample with the participation of experts from other countries, which is expected to reinforce the recommendations of this group of experts.

The variability in the results reflects aspects in which there is not clear consensus yet, which offers new hypotheses to assess.

## Supplementary Information

Below is the link to the electronic supplementary material.

Supplementary file1 (DOCX 28 KB)

## Data Availability

Not applicable.
