# Supplementary material for: Delphi consensus on the diagnosis and treatment of patients with short stature in Spain: GROW-SENS study
Source: J Endocrinol Invest. 2021 Nov 17;45(4):887–97. doi: 10.1007/s40618-021-01696-0 (PMC8918130; doi:10.1007/s40618-021-01696-0)
Supplement: Supplementary file 1 — Supplementary file1 (DOCX 28 KB) [file 40618_2021_1696_MOESM1_ESM.docx]

**ANNEX - Panel of participating experts who completed at least one round (n=43)**

| **Álava** | Díez López, Ignacio |
| --- | --- |
| **Albacete** | Ruiz Cano, Rafael |
| **Alicante** | Aleixandre Blanquer, Fernando |
|  | Fuentes Castello, Miguel Ángel |
|  | Ruiz Pérez, Lorea |
| **Asturias** | Riaño Galán, Isolina |
| **Badajoz** | Arroyo Díez, Francisco Javier |
| **Barcelona** | Bel Comos, Joan Bautista |
|  | Borras Pérez, María Victoria |
|  | Ramón Krauel, Marta |
|  | Yeste Fernández, Diego |
| **Cadiz** | Lechuga Sancho, Alfonso María |
|  | Santos Mata, María Ángeles |
| **Ciudad real** | Palomo Atance, Enrique |
| **Girona** | López Bermejo, Abel |
| **Granada** | Hoyos Gurrea, Raúl |
| **Guadalajara** | Alija Merillas, María Jesús |
| **La Coruña** | Cabanas Rodríguez, Paloma |
|  | Castro Feijoo, Lidia |
| **Las Palmas de Gran Canaria** | Quinteiro González, Sofía |
| **Lleida** | Bosch Muñoz, Jordi |
| **Madrid** | García Cuartero, Beatriz |
|  | Guerrero Fernández, Julio |
|  | Pozo Román, Jesús |
|  | Ramírez Fernández, Joaquín |
|  | Rodríguez Arnao, María Dolores |
|  | Rodríguez Sánchez, Amparo |
|  | Ros Pérez, Purificación |
|  | Sánchez Pozo, Jaime |
| **Murcia** | Donate Legaz, José María |
|  | Escribano Muñoz, Arantxa |
|  | Martos Tello, José María |
| **Navarra** | Chueca Guindulain, María Jesús |
| **Pontevedra** | Chamorro Martin, José Luis |
| **Salamanca** | Prieto Matos, Pablo |
| **Seville** | Bermúdez De la Vega, José Antonio |
|  | Espino Aguilar, Rafael |
| **Valencia** | Alcon Sáez, José Juan |
|  | León Cariñena, Sara |
| **Vizcaya** | Rica Echevarría, Itxaso |
| **Zaragoza** | Bueno Lozano, Gloria |
|  | De Arriba Muñoz, Antonio |
|  | Ferrer Lozano, Marta |
